# Why do students lose the joy of learning? Evidence from engagement, curiosity, and classroom experience

**DOI:** 10.3389/fpsyg.2026.1776932

**Published:** 2026-04-17

**Authors:** Linqiang Wang, Xiaohuan Chen, Lu Sun, Chunming Chen

**Affiliations:** 1 School of Tourism, Shandong Women’s University, Jinan, Shandong, China; 2 College of International Studies, Southwest University, Chongqing, China; 3 Accounting School, Nanfang College, Guangzhou, China; 4 School of Food and Health, Guangzhou City Polytechnic, Guangzhou, Guangdong, China

**Keywords:** classroom experience, curiosity, learning engagement, learning enjoyment, Self-Determination Theory, social cognitive theory, structural equation modeling, Task Value Theory

## Abstract

Learning enjoyment among university students has shown signs of decline in recent years, raising growing concerns about student motivation and engagement in higher education. Although previous research has identified several factors influencing students’ learning experiences, such as learning engagement, curiosity, and classroom environment, most studies have examined these variables independently, with limited attention to how they interact to shape learning enjoyment within an integrated framework. To address this gap, this study adopts a quantitative research design using a questionnaire survey and structural equation modeling (SEM) to investigate the relationships among learning engagement, curiosity, classroom experience, and learning enjoyment. Data were collected from 350 university students representing different academic disciplines and year levels. The results indicate that learning engagement, curiosity, and classroom experience all positively predict learning enjoyment, with classroom experience demonstrating the strongest effect, highlighting the importance of supportive classroom environments and effective teacher–student interactions. In addition, classroom experience moderates the relationship between curiosity and learning enjoyment by strengthening students’ intrinsic motivation and engagement with learning activities. Multi-group analysis further reveals disciplinary differences, showing that the relationships among engagement, classroom experience, and learning enjoyment are stronger among science students than among humanities students. By integrating Self-Determination Theory, Social Cognitive Theory, and Task Value Theory, this study provides a multidimensional explanation of how motivational and contextual factors interact to shape students’ learning enjoyment. The findings offer both theoretical insights and practical implications for improving classroom design, strengthening teacher support, and promoting student engagement in higher education.

## 1 Introduction

### 1.1 The loss of learning enjoyment among students in the context of globalization: a phenomenon and its causes

With the rapid pace of globalization, a growing number of students worldwide are experiencing a decline in their enjoyment of studies. Carini et al. (2006) demonstrate a significant positive correlation between student engagement and academic performance. When students disengage, their academic achievements tend to decline, and they also lose interest in academic activities. In particular, within the context of globalized education, students encounter challenges stemming from diverse educational systems. This growing loss of motivation and enjoyment in learning has become a major focus in educational reform across countries. Hari Rajan et al. (2024) explore how the transition from offline to online learning environments has influenced students’ motivation. Their study reveals that although the expansion of online learning opportunities and technological advancements have created new avenues for education, they have also resulted in a divergence in students’ learning motivation. Specifically, online learning, which lacks face-to-face interactions, has not effectively fostered students’ emotional engagement and interest, leading to a significant reduction in both enjoyment and enthusiasm for learning. Trowler (2010) extends this argument by emphasizing the global nature of the decline in student engagement. He notes that this issue is not confined to particular countries or regions but is observable across various educational systems worldwide. Trowler’s research suggests that, despite ongoing educational reforms and the introduction of new curricula, student engagement remains difficult to sustain, particularly in environments lacking adequate emotional support and interaction. Stenalt et al. (2025) investigate the effects of interdisciplinary educational reforms on students’ learning enjoyment. They argue that, while global educational reforms have promoted interdisciplinary models, they have inadvertently led to a decline in student interest, particularly when classroom interactions are insufficient and when there is a lack of collaboration among students. Thus, balancing the breadth of educational content with students’ emotional involvement has become a critical issue in modern educational reform. Wang (2025) argues that the phenomenon of declining student enjoyment in learning, within the context of globalization, cannot be attributed solely to unappealing course content. It is closely tied to broader factors, including educational systems, cultural expectations, and societal pressures. Wang’s research highlights that learning enjoyment is significantly shaped by the social, cultural, and institutional environments. Particularly in educational systems that overemphasize exam results and standardized testing, students’ intrinsic interests and motivation are often neglected, further exacerbating the loss of enjoyment in learning.

### 1.2 Theoretical development of learning engagement, curiosity, and classroom experience: from traditional motivation models to modern multidimensional perspectives

Learning engagement, curiosity, and classroom experience are pivotal elements that shape students’ motivation and academic success. Early frameworks, such as Self-Determination Theory (SDT) proposed by Deci and Ryan (1985) and further developed by Ryan and Deci (2000), laid the foundation for understanding student motivation in educational contexts. SDT distinguishes between intrinsic and extrinsic motivation, with research demonstrating that intrinsic motivation—driven by personal interest and value—has a more profound effect on learning engagement and academic performance than extrinsic factors like rewards or grades. This distinction became a central tenet for further studies on learning engagement and the enjoyment derived from learning. As the field evolved, Fredricks et al. (2004) introduced a groundbreaking three-dimensional model of learning engagement, breaking it down into behavioral, emotional, and cognitive dimensions. Their work shed light on how these distinct forms of engagement interact to influence academic achievement. Particularly, they underscored the significant role that emotional and cognitive engagement play in shaping students’ learning processes, suggesting that fostering both emotional connections and cognitive investment is essential for enhancing learning outcomes and enjoyment. Building on this foundation, Hidi and Renninger (2006) proposed a four-phase model of interest development, which added depth to the understanding of engagement, especially the role of interest. They traced the progression of interest from situational to individual, emphasizing how students’ evolving interest in learning activities significantly boosts engagement and academic success. Their research highlighted the transformative power of interest in sustaining long-term motivation, underlining that students’ ability to deepen their engagement relies on the growth of their personal connection to the material. Boekaerts (2016) further expanded on the idea of learning engagement, arguing that it is not simply a product of motivation but an integral component of the learning process itself. She particularly focused on the interaction between emotional and cognitive engagement, asserting that the quality of classroom interactions, the emotional support provided by teachers, and thoughtful task design are crucial factors that impact students’ engagement. For effective curriculum design, Boekaerts suggested that educators must consider these factors to cultivate a supportive and engaging learning environment. Finally, Eccles (2016) provided a comprehensive summary of the state of research on learning engagement and motivation, advocating for a broader perspective that views engagement as a dynamic and multidimensional process. She argued that learning engagement is influenced not only by individual differences but also by the educational environment, cultural contexts, and policies. Eccles emphasized the need for future research to explore these multilevel influences more thoroughly, particularly the impact of diverse educational settings and the social and cultural forces that shape students’ engagement and motivation.

### 1.3 Limitations in existing research and the present study’s contribution: an empirical analysis based on a multidimensional model

Despite the substantial contributions of existing research in the fields of learning motivation and engagement, significant gaps remain in our understanding of the dynamic relationships among learning engagement, curiosity, and classroom experience. Hidi and Renninger’s (2006) model of interest development emphasizes the essential role of interest in student learning, proposing a four-stage progression from situational to individual interest, which directly influences learning engagement. However, this model has yet to fully address the complex interactions between interest and students’ emotional and cognitive engagement, particularly within the context of classroom dynamics and students’ self-efficacy. Expanding upon earlier work, Skinner and Pitzer (2012) advanced our understanding of student engagement by emphasizing the importance of emotional responses and resilience in the learning process. While their research sheds light on coping strategies, it predominantly focuses on individual emotional responses and lacks a comprehensive investigation into how classroom experiences, including teacher feedback and peer interactions, influence learning motivation and academic achievement. In addition, Rotgans and Schmidt (2014) explored the relationship between situational interest and the desire for knowledge, noting the significant role of situational interest in fostering engagement. However, their research still falls short of exploring the interactions between situational interest and other dimensions of engagement, such as cognitive and behavioral involvement. This highlights a broader gap in research, where studies typically isolate individual factors but fail to examine the intricate, interconnected effects of multiple engagement dimensions on learning outcomes. As discussed in Section 1.2, Self-Determination Theory (SDT) emphasizes the distinction between intrinsic and extrinsic motivation and highlights the important role of intrinsic motivation in promoting learning engagement and academic achievement. While SDT has been widely applied, few studies have integrated classroom environment factors—such as emotional support, teacher feedback, and peer interaction—into this framework to provide a more holistic view of how these elements collectively influence learning enjoyment. To address this gap, the multidimensional model proposed in this study combines elements from SDT, situational interest, and classroom experience to investigate how these factors interact to shape students’ learning motivation and enjoyment, thus advancing both theoretical and empirical understanding in this area. Finally, while Harackiewicz et al. (2016) highlighted the positive impact of interest cultivation on academic achievement, their research predominantly focuses on the development of interest itself, without considering the ways in which interest interacts with emotional support and classroom interaction. This study’s innovation lies in its integration of existing theoretical frameworks and empirical data to explore how learning engagement, curiosity, and classroom experience intersect, thereby contributing to the erosion of students’ learning enjoyment. By identifying these intersecting factors, this study offers practical intervention pathways and strategies for enhancing educational practices and improving student engagement in the classroom.

## 2 Literature review

### 2.1 The phenomenon and influencing factors of the loss of learning enjoyment among university students

The phenomenon of university students losing their enjoyment in learning has become a widely discussed issue, especially in today’s educational landscape, where academic pressure and external evaluations are intensifying. As a result, intrinsic motivation and emotional engagement often suffer, contributing to a decline in students’ overall learning experiences. Martin (2014) underscored the strong link between students’ interpersonal relationships and their academic success. He specifically highlighted that the quality of teacher-student interactions is a crucial factor in fostering students’ learning enjoyment. Martin’s research suggests that when students receive emotional support from their teachers and are part of a warm, supportive classroom environment, they are more likely to maintain high levels of motivation and positive learning experiences. On the other hand, when emotional support and interaction are lacking in the classroom, students are at risk of losing interest in their studies. Hence, the quality of these interpersonal relationships, particularly teacher-student interactions, plays a pivotal role in both students’ academic development and their enjoyment of learning. In examining learning motivation and emotional engagement, Xie et al. (2025) conducted a thorough analysis that examined the profound impact of academic emotions on students’ learning outcomes. Their findings indicate that emotional fluctuations—particularly negative emotions like anxiety and stress—can significantly diminish students’ motivation and erode their enjoyment of learning. In academic settings that are high-pressure, students’ emotional engagement tends to fluctuate, which not only affects their motivation but also leads to a decline in their overall learning enjoyment. Thus, managing academic emotions and fostering emotional regulation are critical for maintaining students’ engagement and improving learning outcomes. The self-regulated learning framework developed by Schunk and Zimmerman (2008) further contributes to our understanding of learning motivation. They emphasized that self-regulation, learning motivation, and emotional engagement work together to shape students’ learning outcomes. According to their model, the interaction between emotional and cognitive factors is essential in sustaining students’ enjoyment of learning. Schunk and Zimmerman proposed that goal-setting, emotional regulation, and the application of effective learning strategies are key components in maintaining learning engagement. However, as the emphasis on standardized testing and external evaluations increases, students often shift their focus to achieving outcomes rather than enjoying the learning process itself, which leads to a gradual loss of learning enjoyment. Kahu (2013), in her investigation of student engagement, further emphasized that learning engagement is not merely a reflection of students’ participation in classroom activities but also includes their emotional and cognitive responses to the learning tasks at hand. Her study illustrated that when emotional and cognitive engagement are insufficient, students may perceive learning tasks as lacking in meaning, which leads to a loss of enjoyment in their studies. Particularly in higher education, where students face increasing academic challenges, emotional support both within and outside the classroom is essential for preserving their motivation and enjoyment. Kahu stressed the need for educational systems to provide more opportunities for autonomous learning while simultaneously focusing on emotional support to help students sustain their interest and engagement in academic tasks. Reeve and Jang (2006) added that teachers are instrumental in shaping students’ learning experiences, particularly in nurturing their autonomy and emotional engagement. Their research demonstrated that teacher support and feedback significantly impact students’ motivation. Specifically, when teachers support students’ autonomy, their engagement and enjoyment of learning are markedly enhanced. In traditional educational environments, however, excessive external control and evaluation often undermine students’ sense of autonomy, which in turn diminishes their interest and engagement in learning. Ketonen et al. (2016) explored the relationship between early-stage student engagement and academic success, revealing that students’ adaptation and academic engagement during their early university years are closely tied to their long-term academic performance. The loss of learning enjoyment is common when students struggle to adjust to the academic environment early on. Ketonen et al. argued that without adequate support and guidance in these critical early stages, students may experience insufficient emotional and cognitive engagement, which in turn hampers their learning enjoyment and academic achievement.

### 2.2 Learning engagement and academic performance: motivation and engagement among university students

The relationship between learning engagement and academic performance has long been a central focus of educational psychology. Academic engagement, a key element in the learning process, is widely recognized for its significant impact on students’ academic outcomes and achievements. Recent studies have increasingly highlighted that learning engagement extends beyond mere classroom behavior to encompass three distinct dimensions: emotional, cognitive, and behavioral engagement. These dimensions interact with one another, collectively influencing students’ academic performance. García-Martínez et al. (2021) explored the mediating role of learning engagement in shaping university students’ academic success and overall quality of life. Their research demonstrates that academic engagement influences academic performance not only by enhancing learning motivation and academic investment but also by contributing to students’ psychological wellbeing. Specifically, their findings reveal that, under stress, increased academic engagement helps alleviate anxiety and boosts students’ self-confidence, thereby improving both their academic outcomes and emotional health. Further expanding on this, Chen et al. (2023) examined the impact of social support on academic engagement. Their study found that social support positively affects students’ life satisfaction and academic motivation, which in turn strengthens their learning engagement. They argued that life satisfaction and academic motivation function as mediators in the relationship between social support and academic engagement, providing important insights for educational practice. This suggests that institutions should prioritize fostering strong social support networks, particularly from family and peers, to enhance students’ academic engagement and motivation. In a related study, Acosta-Gonzaga (2023) investigated the role of self-esteem in academic engagement, highlighting the significant influence of self-esteem on students’ learning involvement. The research found that self-esteem plays a crucial role in promoting academic engagement, particularly when students have high academic self-efficacy and self-belief. Acosta-Gonzaga’s findings further suggest that self-esteem indirectly enhances academic performance by bolstering students’ learning motivation. This emphasizes the importance of psychological factors, such as self-esteem and self-efficacy, as intrinsic motivators of academic achievement and learning enjoyment. In the context of online learning, Akram and Li (2024) explored how teacher-student relationships impact online learning engagement. They discovered that emotional support, feedback, and interaction from teachers significantly improve students’ academic motivation and engagement in online settings. Their study indicates that teacher autonomy support plays a vital role in fostering students’ enthusiasm and active participation in online learning environments. In particular, in the absence of face-to-face interactions, teachers’ emotional support addresses students’ emotional needs and enhances their learning motivation. This research provides crucial theoretical support for understanding the impact of teacher behavior and interaction patterns in online education. Moreover, Meng and Zhang (2023) analyzed the role of academic self-efficacy in shaping academic performance, particularly its mediating role in academic engagement. Their study found that academic self-efficacy strongly influences academic performance by enhancing students’ engagement in learning. This suggests that students’ belief in their own academic abilities is a key factor in regulating their motivation and participation in learning activities, ultimately affecting their academic performance. These findings provide robust empirical evidence for understanding the relationship between academic self-efficacy and learning motivation. Finally, Tannoubi et al. (2025) examined the influence of academic engagement on academic success, focusing on university students majoring in physical education. Their research revealed that the interaction between effective learning methods and academic engagement significantly impacts academic performance. The study showed that academic engagement promotes the adoption of effective learning strategies, which in turn positively influences academic outcomes. This research offers practical insights into how educators can enhance students’ learning methods and engagement to improve their academic success.

### 2.3 The impact of curiosity and classroom experience on university students’ learning enjoyment

Curiosity and classroom experience are integral to students’ learning motivation, playing a pivotal role in shaping both their learning enjoyment and academic performance. In recent years, it has become increasingly evident that curiosity not only influences students’ engagement in learning but also serves as a key regulator of their academic performance through its impact on emotional and cognitive engagement. Linnenbrink-Garcia et al. (2012) explored the antecedents and outcomes of situational interest, emphasizing its importance in the learning process. Their findings demonstrated that situational interest not only enhances learning enjoyment by promoting engagement but also positively influences academic performance by fostering students’ intrinsic motivation. While the effect of situational interest tends to be immediate and transient, it has the potential to evolve into long-term learning interest, thereby contributing to sustained learning enjoyment. In the context of curiosity, Ye et al. (2022) examined the role of situational interest within classroom learning environments. Their study, which integrated both experimental and naturalistic approaches, revealed the direct impact of situational interest on student learning outcomes. They found that situational interest not only improves students’ attention and engagement in the classroom but also deepens their interest in the subject matter being taught. This study underscores the significant role that the classroom environment and teaching strategies play in stimulating students’ situational interest. A comprehensive review by Guo and Fryer (2024) further elaborated on the diverse sources of situational interest, identifying six major sources and discussing their practical applications in educational contexts. They emphasized that factors such as teaching methods, classroom interaction, and the engaging nature of course content are central to stimulating students’ situational interest. These elements, in turn, enhance students’ curiosity and learning engagement, which collectively contribute to greater learning enjoyment. Additionally, Rotgans and Schmidt’s (2017) interest development theory suggests that the stimulation of situational interest not only boosts immediate engagement but also fosters long-term learning motivation by deepening individual interest in the subject. Their research found that the initial activation of situational interest is crucial in sustaining long-term engagement and academic achievement. Lobo (2024) further explored how curiosity influences learning engagement, particularly in physical education courses such as gymnastics. Her research indicated that students’ curiosity plays a critical role in increasing their learning motivation, as it encourages classroom participation and engagement by introducing challenges and enjoyment into the curriculum. This finding highlights the reciprocal relationship between curiosity and learning engagement, particularly in hands-on courses that require physical involvement, where curiosity has a pronounced positive impact on learning outcomes. Murayama et al. (2019) proposed a dynamic relationship between curiosity and interest from the perspective of reward learning. They argued that curiosity stimulates students’ intrinsic motivation and enhances their engagement with learning content through the reward learning process. Their findings underscore that the activation of curiosity not only strengthens cognitive engagement but also boosts long-term learning motivation, thereby improving academic achievement and enhancing learning enjoyment. In a related study, Zeng et al. (2025) explored how curiosity in the classroom fosters students’ creativity. Their research found that curiosity significantly promotes students’ creative thinking and problem-solving skills, particularly in subjects that require critical thinking and creative approaches. By stimulating curiosity, students’ learning enjoyment is significantly enhanced, as it deepens both engagement and academic satisfaction.

### 2.4 How to address the loss of learning enjoyment among university students: pathways for psychological and educational interventions

The loss of learning enjoyment among university students, particularly in the context of mounting academic pressure and external evaluations, has emerged as a critical issue in global higher education. Existing literature suggests that both psychological and educational interventions have significant effects on students’ learning motivation, engagement, and overall enjoyment. Tao et al. (2022) conducted a meta-analysis that highlighted the crucial role of teacher support in students’ academic engagement and performance. Their research demonstrated that when students perceive emotional support from their teachers, it positively influences their learning engagement and academic performance. This, in turn, helps mitigate the loss of learning enjoyment, especially under the strain of academic pressure. In terms of curriculum design, Wijnia et al. (2024) explored the impact of problem-based, project-based, and case-based learning models on students’ intrinsic motivation. Their research found that these instructional models, particularly problem-based and project-based learning, significantly enhance students’ intrinsic motivation and learning enjoyment by fostering autonomy and improving problem-solving skills. These models stimulate students’ curiosity and engagement, which not only boosts learning motivation but also contributes to better academic performance. Ng et al. (2025) extended this research by proposing that low-intensity positive education, which enhances students’ sense of civic engagement, can improve mental health and help restore academic engagement and learning enjoyment. Their study shows that multidimensional interventions can strengthen students’ psychological resilience, self-confidence, and motivation, thus reducing the loss of learning enjoyment. Shen et al. (2024) further investigated how teacher emotional support influences academic engagement. They found that positive academic emotions and mastery goals promoted by teacher support help students maintain their engagement and interest in academic tasks, leading to improved academic performance and enhanced learning enjoyment. The global pandemic has also influenced educational practices, as demonstrated by Xu and Zou (2023), who examined the impact of online teaching on university foreign language students’ learning performance. Their research highlighted that appropriate online teaching interventions, including interactive and challenging course designs, can significantly enhance academic performance and reduce the loss of learning enjoyment in online learning environments. These findings suggest that even in virtual settings, fostering student engagement through effective teaching strategies remains vital. In addition, Simón-Grábalos et al. (2025) conducted a systematic review to explore the effects of self-regulated learning interventions on students’ academic performance and engagement. Their study found that interventions designed to promote self-regulated learning, especially among first-year university students, significantly improved learning engagement and academic self-confidence. This research underscores the importance of helping students manage their emotions and motivation throughout the learning process, preventing the loss of learning enjoyment. While existing interventions provide a valuable theoretical foundation for enhancing students’ learning motivation and engagement, few studies have integrated learning engagement, curiosity, and classroom experience into a comprehensive, multidimensional model to explore their combined impact on learning enjoyment. The innovation of this study lies in proposing an empirical model that combines these factors to investigate their interactive effects on the loss of learning enjoyment among university students. This research not only empirically validates the role of these interconnected factors but also suggests effective educational interventions, offering new insights and strategies for restoring learning enjoyment and enhancing student motivation in higher education.

## 3 Materials and methods

### 3.1 Research design and theoretical framework

This study employs a quantitative research design, combining a questionnaire survey with structural equation modeling (SEM) to systematically explore the impact of learning engagement, curiosity, and classroom experience on learning enjoyment, and to examine the mechanisms underlying their interactions. Data were collected through an online survey, which yielded 350 valid responses from students across different academic disciplines, gender groups, and year levels, ensuring a diverse and representative sample. The key variables in this study include learning engagement, curiosity, classroom experience, and learning enjoyment, all of which were measured using validated scales: the Academic Engagement Scale for learning engagement, the Curiosity Scale for curiosity, the Classroom Climate Scale for classroom experience, and a custom scale for learning enjoyment, which considers dimensions such as interest, enjoyment, challenge, achievement, and self-efficacy. The reliability and validity of these scales have been well established in prior research, confirming the consistency and accuracy of the data. For data analysis, this study utilized structural equation modeling (SEM), a method well-suited to assess both the direct and indirect relationships among multiple independent and dependent variables. AMOS 24.0 software was used to conduct the analysis, with maximum likelihood estimation (MLE) employed to estimate the model parameters. The model fit was evaluated using standard fit indices, including χ^2^/df, CFI, TLI, RMSEA, and SRMR. To further examine the significance of mediating effects and uncover the underlying mechanisms among variables, the Bootstrap method was applied. Additionally, multi-group SEM analysis was conducted to assess path differences across academic disciplines and gender groups, providing deeper insights into potential variations within these subgroups. In terms of the theoretical framework, this study integrates Self-Determination Theory (SDT), Social Cognitive Theory (SCT), and Task Value Theory. SDT underscores the central role of intrinsic motivation in fostering learning engagement, suggesting that learning engagement and curiosity enhance learning enjoyment by fulfilling students’ basic psychological needs for autonomy, competence, and relatedness. SCT emphasizes the interaction between individuals, their environment, and their behaviors, proposing that classroom experience influences students’ learning motivation and enjoyment by enhancing their self-efficacy and providing emotional support. Task Value Theory, in turn, argues that students’ perceptions of the value and challenge of learning tasks directly affect their engagement and enjoyment. According to this theory, classroom experience, as an external environmental factor, shapes students’ evaluation of task value and challenge, influencing both their engagement and enjoyment in the learning process. By integrating these three theories, this study constructs a multidimensional theoretical framework that aims to deepen our understanding of how learning engagement, curiosity, and classroom experience interact to foster students’ learning enjoyment. This framework not only supports the research hypotheses but also provides a robust foundation for the subsequent data analysis. The SEM analysis enables the study to precisely measure the relationships between the variables, thereby validating the effectiveness of the theoretical framework and contributing valuable theoretical support for future research in the field of educational psychology.

### 3.2 Measurement instruments

To ensure the reliability and validity of the constructs, all variables in this study were measured using previously validated instruments widely applied in educational and psychological research. The questionnaire consisted of four sections measuring learning engagement, curiosity, classroom experience, and learning enjoyment. All items were rated on a five-point Likert scale ranging from 1 (strongly disagree) to 5 (strongly agree). The instruments, their sources, and example assessment items used to measure each construct are described below.

#### 3.2.1 Learning engagement

Learning engagement was measured using the Academic Engagement Scale adapted from the three-dimensional engagement framework proposed by Fredricks et al. (2004). The scale assesses students’ behavioral, emotional, and cognitive engagement in learning activities. Previous studies have reported strong psychometric properties for this scale in higher education contexts. In the present study, the scale consisted of items assessing students’ active participation, emotional involvement, and cognitive investment in learning. Example items used to assess learning engagement include: “I actively participate in classroom learning activities” and “I try to understand the course material deeply rather than simply memorize it.”

#### 3.2.2 Curiosity

Curiosity was measured using a scale adapted from prior research on epistemic curiosity and learning motivation (e.g., Kashdan et al., 2009). The instrument captures students’ tendency to explore new knowledge, ask questions, and seek intellectual challenges during learning. Previous studies have demonstrated satisfactory reliability and construct validity for this scale in educational settings. Example items used to evaluate curiosity include: “I enjoy exploring new ideas even when they are challenging” and “I feel excited when I encounter unfamiliar topics in class.”

#### 3.2.3 Classroom experience

Classroom experience was assessed using items adapted from the Classroom Climate Scale developed in learning environment research (Fraser, 2012). The scale evaluates students’ perceptions of classroom atmosphere, teacher support, and interaction within the learning environment. Prior studies have confirmed the reliability and validity of this instrument across different educational contexts. Example items include: “The teacher provides helpful feedback during classroom activities” and “The classroom environment encourages discussion and interaction.”

#### 3.2.4 Learning enjoyment

Learning enjoyment was measured using a customized scale developed for this study based on the academic emotions framework proposed by Pekrun (2006). The scale captures students’ positive emotional experiences during learning, including interest, enjoyment, challenge, and perceived competence. The development of the scale followed standard questionnaire design procedures, and its reliability and validity were examined in the present sample through Cronbach’s alpha, composite reliability (CR), and average variance extracted (AVE). Example items include: “I feel enjoyment when learning new knowledge in my courses” and “Learning activities in this course make me feel interested and motivated.”

### 3.3 Indicator system

**FIGURE 1:**
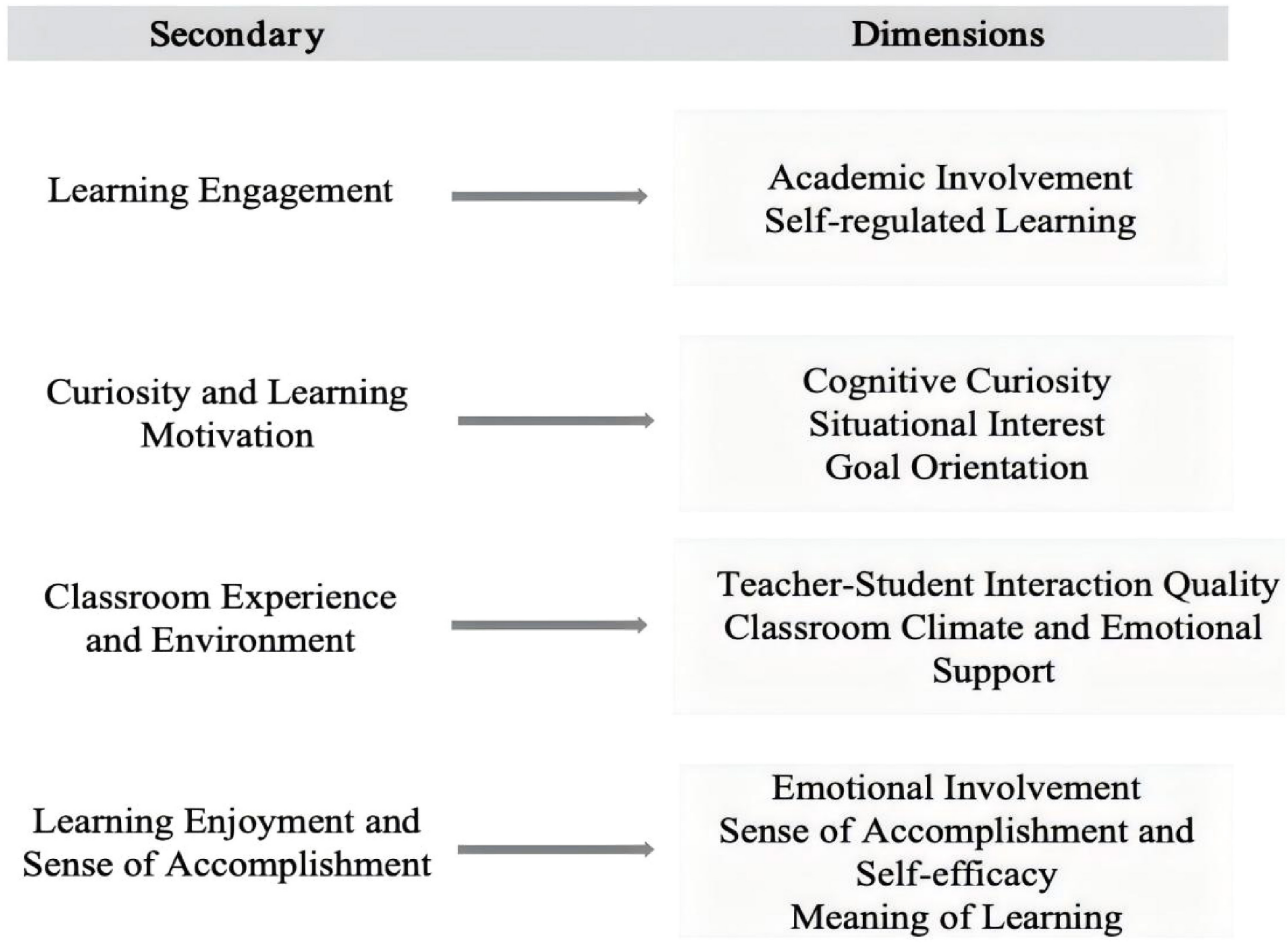
The indicator system for learning engagement, curiosity, classroom experience, and learning enjoyment.

Figure 1 presents the indicator system for the four core domains examined in this study: learning engagement, curiosity and learning motivation, classroom experience and environment, and learning enjoyment and sense of accomplishment. Learning engagement is represented by academic involvement and self-regulated learning. Curiosity and learning motivation includes cognitive curiosity, situational interest, and goal orientation. Classroom experience and environment is reflected in teacher–student interaction quality and classroom climate and emotional support. Learning enjoyment and sense of accomplishment is represented by emotional involvement, sense of accomplishment and self-efficacy, and meaning of learning. This indicator system provides a structured foundation for variable operationalization and subsequent empirical analysis.

## 4 Results

### 4.1 Measurement tools reliability and validity analysis: constructing an efficient learning experience evaluation model

Table 1 presents the reliability and validity of the measurement tools used in this study. Notably, the constructs of learning engagement and learning enjoyment show strong results, with standardized loadings and Cronbach’s α values indicating stability and effectiveness in measurement. The Cronbach’s α value for classroom experience is 0.823, which indicates acceptable internal consistency and suggests that the measurement of this construct is reliable. At the same time, classroom experience represents a relatively complex and multidimensional construct that involves classroom interaction, emotional support, and the overall learning environment. These characteristics highlight a long-standing challenge in educational measurement: although measurement tools continue to improve, capturing the full range of multidimensional constructs such as classroom experience remains inherently complex. For the classroom experience construct—encompassing teacher–student interactions, emotional support, and the learning atmosphere—there are likely contextual variations in its impact across different cultural and educational settings. This variability reflects the contextual nature of classroom experience and suggests that students’ perceptions of classroom environments may differ depending on institutional and cultural contexts. While the measurement tools for learning engagement and learning enjoyment show strong reliability, the measurement of classroom experience may involve broader contextual factors related to classroom dynamics and interaction patterns. Given these considerations, future research may explore the integration of both qualitative and quantitative methods to provide a more precise and nuanced understanding of these constructs in diverse educational contexts, thereby offering deeper insights into the factors influencing learning enjoyment and engagement, particularly in educational settings characterized by diverse cultural and emotional dynamics.

**TABLE 1:** Measurement tools reliability and validity analysis.

Construct	Standardized loading (λ)	*t*-value	Cronbach’s α	CR	AVE
Learning_engagement	0.482	7.68	0.845	0.876	0.725
Curiosity	0.532	8.21	0.836	0.889	0.738
Classroom_experience	0.512	7.99	0.823	0.862	0.719
Learning_enjoyment	0.67	9.1	0.86	0.903	0.783

### 4.2 Descriptive statistics of sample characteristics and core variables

Table 2 provides an overview of the distribution and variability of the core variables. The mean score for learning engagement is 3.65, with a standard deviation of 1.02, suggesting that most students show moderate levels of participation in classroom activities. However, substantial individual variation is observed, indicating that students exhibit different degrees of initiative, which may be influenced by factors such as the classroom environment, teacher-student interaction, and individual motivation. For curiosity, the mean score is 3.58, accompanied by a standard deviation of 1.05, reflecting a general interest in exploring new knowledge among students. Yet, the wide variation in scores indicates that some students are more motivated than others to actively engage with learning materials, a difference that could stem from factors such as course content design and the teaching methods employed. The mean score for classroom experience is 3.60, with a standard deviation of 1.01, showing that students generally have a positive perception of their classroom environment. However, the data reveal some variability, with certain students perceiving a lack of interaction or emotional support in the classroom. This gap may have direct implications for their engagement and overall learning experience. Lastly, the mean score for learning enjoyment is 3.52, with a standard deviation of 1.03. This indicates that while students experience some enjoyment in the learning process, significant variation exists in how they perceive this enjoyment. Several external factors, including academic pressure, external evaluations, and the classroom atmosphere, likely contribute to this disparity. Students who face high levels of academic pressure or those who feel constrained by external evaluation systems might find it difficult to derive enjoyment from learning, which in turn affects their engagement and motivation.

**TABLE 2:** Descriptive statistics of core variables.

Variable	Mean	Standard deviation	Min	25th percentile	Median	75th percentile	Max
Learning_engagement	3.65	1.02	1	3	4	5	5
Curiosity	3.58	1.05	1	3	4	5	5
Classroom_experience	3.60	1.01	1	3	4	5	5
Learning_enjoyment	3.52	1.03	1	3	4	5	5

### 4.3 Correlation between learning engagement and learning enjoyment

Table 3 provides insights into the correlations among learning engagement, curiosity, classroom experience, and learning enjoyment. The correlation between learning engagement and learning enjoyment is 0.68, which indicates a strong positive relationship between students’ active participation in the classroom and the enjoyment they derive from the learning process. This finding suggests that fostering learning engagement can promote enjoyment, but it is important to note that such engagement alone does not guarantee enhanced learning enjoyment. Classroom interaction, teacher emotional support, and the appeal of the course content also play pivotal roles in this dynamic. The correlation between learning engagement and curiosity stands at 0.58, pointing to a moderate but meaningful relationship between students’ engagement in learning and their desire to explore new knowledge. While this correlation is statistically significant, it does not imply that increased engagement will universally spark curiosity in all students. The variability in students’ responses to different learning activities could be influenced by factors such as the level of challenge in course content and the teacher’s ability to create an engaging learning environment. Therefore, educators should consider how to design courses and teaching strategies that stimulate curiosity, thereby fostering greater engagement in the learning process. Classroom experience demonstrates generally strong correlations with the other variables, particularly learning enjoyment (0.70) and learning engagement (0.60). This suggests that the quality of the classroom environment, including teacher-student interactions and emotional support, significantly influences students’ engagement and emotional experiences. Classroom experience is multifaceted, encompassing both the physical classroom environment and the emotional atmosphere created by interactions between students and teachers. However, the complexity of this construct means that it cannot be fully captured by a single-dimensional measure. The challenge remains in developing comprehensive tools that effectively assess all aspects of classroom experience in educational research. The correlation analysis in Table 3 reveals strong positive relationships among the variables of learning engagement, curiosity, classroom experience, and learning enjoyment. However, the relationships behind these correlations are influenced by multiple factors, including the classroom environment, teaching methods, and individual student characteristics. Consequently, educational practice should emphasize a multidimensional approach, particularly in the design of classroom experiences and instructional strategies. By addressing students’ emotional needs, fostering their curiosity, and ensuring robust external support, educators can create an environment that maximizes both engagement and learning enjoyment.

**TABLE 3:** Correlation matrix of core variables.

Variable	Learning_engagement (LE)	Curiosity (CU)	Classroom_experience (CE)	Learning_enjoyment (LEJ)
Learning_engagement (LE)	1	0.58**	0.60**	0.68**
Curiosity (CU)	0.58**	1	0.59**	0.63**
Classroom_experience (CE)	0.60**	0.59**	1	0.70**
Learning_enjoyment (LEJ)	0.68**	0.63**	0.70**	1

**p < 0.01.

### 4.4 Predictive power of learning engagement, curiosity, and classroom experience on learning enjoyment

**FIGURE 2:**
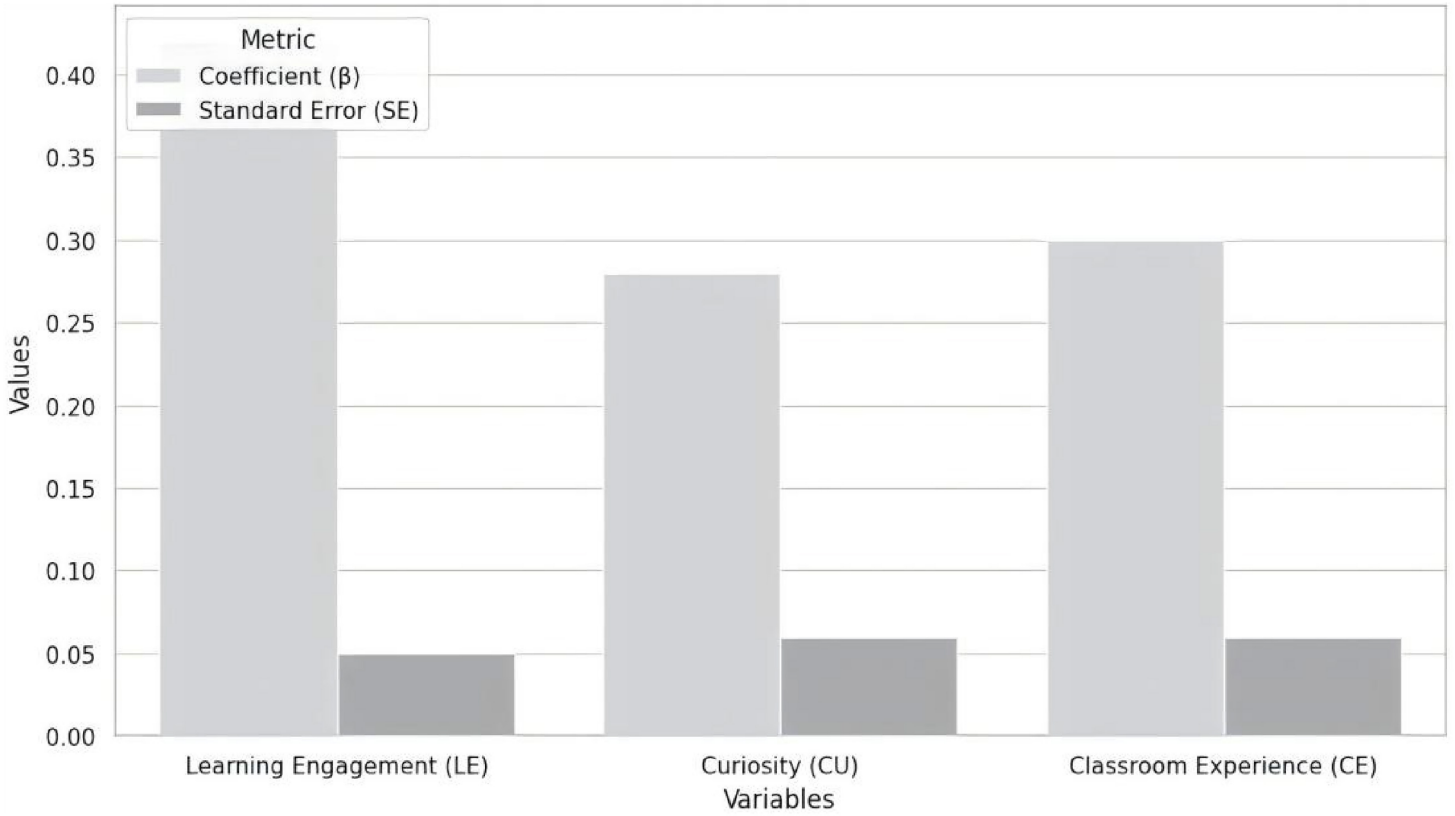
Bar plot of predictors of learning enjoyment: coefficients and standard errors for learning engagement, curiosity, and classroom experience.

Regression analysis presented in Figure 2 and Table 4 shows that learning engagement, curiosity, and classroom experience all significantly predict learning enjoyment. The regression coefficient for learning engagement is 0.42, with a standard error of 0.05, a *t*-value of 8.00, and a *p*-value of 0.000, indicating a strong positive relationship between students’ learning engagement and their learning enjoyment. This highlights the importance of students’ active involvement in the learning process. However, this also prompts further reflection on the connection between learning engagement and learning enjoyment: although learning engagement has a significant effect, its influence is likely shaped by other factors, such as the classroom environment, instructional design, and interactions between students and instructors. This suggests that learning engagement is one of several contributing factors to learning enjoyment, rather than the only determinant. The regression coefficient for curiosity is 0.28, with a standard error of 0.06, a *t*-value of 4.67, and a *p*-value of 0.000, suggesting that curiosity has a moderate, yet statistically significant, impact on learning enjoyment. This indicates that students’ desire to explore new knowledge plays a role in enhancing their learning experience. However, the effect of curiosity appears to be secondary to that of learning engagement, likely because curiosity requires specific classroom contexts and engaging teaching materials to be fully stimulated. Simply presenting learning content may not be enough to effectively spark students’ curiosity. The regression coefficient for classroom experience is 0.30, with a standard error of 0.06, a *t*-value of 5.00, and a *p*-value of 0.000, further supporting the substantial influence of the classroom environment on students’ learning enjoyment, particularly the quality of interactions and emotional support provided within the classroom. This finding emphasizes that learning enjoyment is not solely driven by students’ intrinsic motivation, but is also shaped by external factors such as teacher support, peer relationships, and the classroom atmosphere. Although the influence of classroom experience is slightly lower than that of learning engagement, it still plays a crucial role in fostering emotional engagement, reflecting the complex interaction between emotional and cognitive factors in education. By synthesizing the data from Figure 2 and Table 4, it becomes clear that while all three factors—learning engagement, curiosity, and classroom experience—are significant predictors of learning enjoyment, the analysis also reveals a complex dynamic in educational practice: enhancing learning enjoyment requires not only students’ active participation but also the support provided by the classroom environment and teachers. Therefore, future educational reforms should place greater emphasis on optimizing classroom experiences and fostering students’ curiosity to further promote learning enjoyment.

**TABLE 4:** Regression analysis: predictors of learning enjoyment.

Variable	Coefficient (β)	Standard error (SE)	*t*-value	*p*-value	Significance
Learning_engagement (LE)	0.42	0.05	8.00	0.000	***
Curiosity (CU)	0.28	0.06	4.67	0.000	***
Classroom_experience (CE)	0.30	0.06	5.00	0.000	***

***p < 0.001.

### 4.5 Causal pathways between learning engagement and learning enjoyment: path analysis and SEM

**FIGURE 3:**
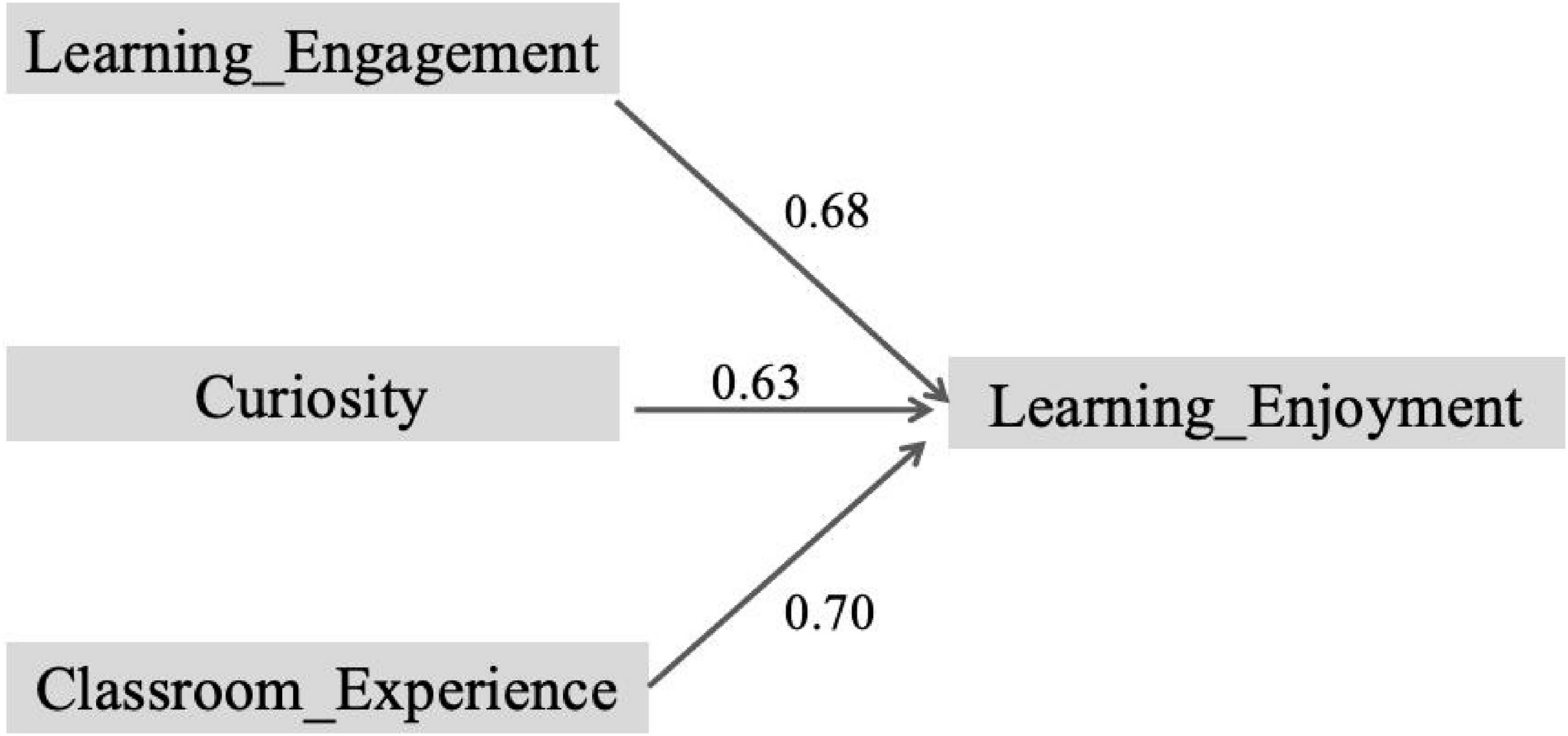
Path diagram of causal pathways between learning engagement, curiosity, classroom experience, and learning enjoyment.

Figure 3 and Table 5 illustrate that learning engagement, curiosity, and classroom experience all predict learning enjoyment. The path coefficient for learning engagement is 0.68, highlighting the significant role of students’ active participation in their learning. This finding underscores the importance of engagement in fostering learning enjoyment. However, it also suggests that increasing engagement alone may not be enough to enhance learning enjoyment; elements such as classroom interaction and teacher support are equally crucial in shaping the learning experience. The path coefficient for curiosity is 0.63, indicating a positive influence on learning enjoyment. While its effect is not as large as that of learning engagement, curiosity still plays a meaningful role in motivating students to explore new knowledge. The somewhat weaker effect of curiosity may be attributed to its dependence on several factors, such as the way classroom tasks are designed, the materials presented, and how effectively teachers can stimulate interest. The path coefficient for classroom experience is 0.70, which points to the dominant influence of classroom environment and teacher-student interactions on learning enjoyment. This finding emphasizes the importance of emotional support and a positive classroom atmosphere in fostering students’ learning experiences. However, the strength of classroom experience is moderated by factors like classroom culture and teaching style, suggesting that the classroom experience is not merely a passive response but involves more complex cognitive and emotional engagement. The analysis presented in Figure 3 and Table 5 reinforces the critical roles that learning engagement, curiosity, and classroom experience play in promoting learning enjoyment. These results point to the complex nature of the educational process and suggest that improving students’ learning enjoyment requires a multifaceted approach, which includes optimizing classroom experiences, designing engaging tasks, and providing emotional support.

**TABLE 5:** Path analysis results: causal pathways in SEM.

Path	Path coefficient (β)	*p*-value	Significance
Learning_engagement → learning_enjoyment	0.68	0.000	***
Curiosity → learning_enjoyment	0.63	0.000	***
Classroom_experience → learning_enjoyment	0.70	0.000	***

***p < 0.001.

### 4.6 Moderating effects of gender and discipline: multi-group analysis

Table 6 presents the moderating effects of gender and academic discipline on learning engagement. The regression coefficient for learning engagement is 0.42 (*p* < 0.001), which emphasizes its significant role in predicting learning enjoyment. However, the path coefficient for gender (male = 0, female = 1) and learning engagement is 0.08 (*t* = 0.57, *p* = 0.571), indicating that gender does not significantly influence learning engagement. This result suggests that although gender is often considered an important factor in educational studies, its effect on learning engagement in this context appears limited. It indicates that the influence of gender on learning engagement may be shaped by cultural contexts and individual differences, and thus should not be the primary basis for educational strategies. In contrast, the effect of academic discipline on learning engagement is significant. The regression coefficient for academic discipline is 0.28 (*t* = 2.33, *p* = 0.02), showing that students in scientific disciplines exhibit higher levels of engagement than their peers in humanities. This could be attributed to the structured nature of scientific studies, including distinct learning methods, assessment standards, and course design. Based on these findings, it is important for educators to tailor their teaching strategies according to the characteristics of each discipline to foster greater engagement. The interaction between gender and academic discipline (0.18, *t* = 1.00, *p* = 0.318) does not reach statistical significance, suggesting that the combined impact of gender and academic discipline on learning engagement is minimal. This suggests that while gender and academic discipline may interact in some cases, the effect of gender on learning engagement is weak in this study, possibly reflecting the decreasing influence of gender differences in contemporary educational systems. Overall, the analysis highlights the complex relationship between gender, academic discipline, and learning engagement. Academic discipline, particularly the characteristics and teaching methods associated with it, appears to play a more significant role in shaping students’ engagement. Educators should consider both the specific features of academic disciplines and individual student differences when designing teaching strategies to enhance learning engagement.

**TABLE 6:** Moderating and discipline.effects of gender

Variable	Coefficient (β)	Standard error (SE)	*t*-value	*p*-value	Significance
Learning_engagement (LE)	0.42	0.05	8.4	0.000	***
Gender (Male = 0, Female = 1)	0.08	0.14	0.57	0.571	
Discipline (Arts = 0, Science = 1)	0.28	0.12	2.33	0.02	**
Gender × Discipline Interaction	0.18	0.18	1.00	0.318	

**p < 0.01, ***p < 0.001.

## 5 Discussion

### 5.1 Exploring the interaction and mechanisms of learning engagement, curiosity, and classroom experience on learning enjoyment

This study highlights the critical roles that learning engagement, curiosity, and classroom experience play in fostering learning enjoyment while also revealing the complex interactions among these factors. The results show that learning engagement has a strong positive effect on learning enjoyment (β = 0.68), emphasizing the importance of students’ active participation and cognitive investment in the learning process. This finding is consistent with the engagement framework proposed by Fredricks et al. (2004), which conceptualizes engagement as a multidimensional construct involving behavioral, emotional, and cognitive components. Their framework suggests that when students actively participate in learning tasks, invest cognitively in understanding course materials, and experience emotional involvement in the learning process, they are more likely to experience positive academic emotions, including enjoyment. The present study supports this theoretical perspective by demonstrating that higher levels of engagement are associated with stronger learning enjoyment ‘’among university students. Curiosity also shows a positive influence on learning enjoyment (β = 0.63), although its effect is slightly weaker than that of learning engagement. This finding aligns with the reward-learning perspective of curiosity proposed by Murayama et al. (2019), which suggests that curiosity functions as an intrinsic motivational mechanism that stimulates knowledge exploration and cognitive engagement when learners encounter information gaps or intellectually stimulating tasks. However, the relatively smaller effect observed in this study suggests that curiosity may be more context-dependent than engagement. In other words, curiosity may only translate into sustained learning enjoyment when instructional materials and learning tasks are sufficiently novel, challenging, and intellectually stimulating. Among the three predictors examined in this study, classroom experience demonstrates the strongest effect on learning enjoyment (β = 0.70), highlighting the central role of classroom climate, teacher support, and interaction quality in shaping students’ learning experiences. This finding is consistent with prior research showing that teacher autonomy support and emotionally supportive classroom environments significantly enhance students’ motivation and engagement (Reeve and Jang, 2006). A supportive classroom environment not only strengthens students’ emotional connection to learning but also encourages active participation and exploration, thereby amplifying the effects of engagement and curiosity. Taken together, these findings suggest that learning enjoyment is not driven by a single factor but rather emerges from the interaction between internal motivational factors and external learning environments. While learning engagement and curiosity represent important internal drivers of motivation, classroom experience provides the contextual support necessary for these motivations to translate into positive learning experiences. This integrated perspective highlights the importance of designing classroom environments that simultaneously stimulate curiosity, promote active engagement, and provide emotional support to students in order to enhance learning enjoyment in higher education.

### 5.2 The moderating effects of gender and academic discipline on learning engagement and learning enjoyment: a multilevel analysis perspective

This study further examines how gender and academic discipline moderate the relationship between learning engagement and learning enjoyment. The results indicate that academic discipline plays a significant role in this relationship, whereas the influence of gender is relatively weak. Specifically, the regression coefficient for academic discipline is 0.28 (*p* < 0.05), suggesting that disciplinary background significantly affects students’ learning engagement and subsequently contributes to their learning enjoyment. This finding is broadly consistent with Kahu (2013), who argued that disciplinary contexts and learning environments strongly shape patterns of student engagement and learning experiences. In general, students in scientific disciplines tend to encounter more structured learning tasks, such as problem-solving activities, laboratory work, and clearly defined learning objectives. These tasks often require sustained cognitive effort, which may encourage deeper engagement and lead to a stronger sense of achievement and enjoyment during the learning process. In contrast, learning tasks in the humanities are often more open-ended and flexible, meaning that students’ engagement may depend more heavily on individual interest and contextual factors, which can result in greater variability in learning engagement and enjoyment. At the same time, the present findings extend previous research by suggesting that disciplinary context may influence not only how students engage with learning tasks but also how engagement translates into emotional learning experiences such as enjoyment. In addition, this study finds that the moderating effect of gender on learning engagement is not significant (β = 0.08, *p* = 0.571), indicating that gender differences in learning motivation and engagement may be diminishing in contemporary higher education settings. This result aligns with the perspective of Reeve and Jang (2006), who emphasized that in autonomy-supportive classroom environments, students’ engagement is more strongly influenced by contextual factors such as teacher support and classroom interaction rather than demographic characteristics such as gender. As higher education increasingly emphasizes personalized learning and educational equity, the role of gender in shaping learning behaviors appears to be gradually weakening. Overall, the findings suggest that students’ learning experiences are shaped by complex contextual factors, and that disciplinary context may exert a stronger influence on learning engagement and enjoyment than demographic variables such as gender. Therefore, instructional strategies should place greater emphasis on disciplinary characteristics and students’ psychological needs in order to effectively promote both learning engagement and learning enjoyment.

### 5.3 The impact of classroom environment and emotional support on learning enjoyment: integration of theory and practice from an interdisciplinary perspective

The results of this study reveal the significant influence that the classroom environment and emotional support have on students’ learning enjoyment, particularly in shaping their motivation. Emotional factors are crucial and should not be overlooked. The regression analysis showed that classroom experience has a coefficient of 0.70, suggesting that the classroom atmosphere and teacher–student interactions are key drivers in enhancing students’ learning enjoyment. These elements influence not only students’ cognitive engagement but also their emotional involvement in learning, highlighting the importance of emotional support throughout the learning process. This finding is consistent with previous research suggesting that classroom environments play an important role in shaping students’ motivation and academic emotions. Prior studies have also emphasized that learning motivation and enjoyment are influenced not only by intrinsic factors but also by external elements, particularly the classroom environment and the emotional support provided by teachers. When students perceive positive feedback and emotional encouragement from their teachers, their motivation and interest in learning tend to increase, which in turn enhances their learning enjoyment. At the same time, the study highlights the complex nature of classroom interactions across different academic disciplines. In science classrooms, teaching activities often focus on structured tasks and problem solving, where teacher–student interactions are centered on knowledge acquisition and application. In contrast, humanities classrooms tend to emphasize discussion and critical thinking, where emotional support and encouragement may play a more visible role. This suggests that emotional support in the classroom is not only about creating a positive atmosphere but also about how teachers use feedback and interaction to respond to students’ needs in different disciplinary contexts. In this sense, the classroom should be understood not simply as a physical space for learning but also as a site of emotional and cognitive exchange. When designing courses, educators should therefore consider how classroom interactions vary across disciplines and aim to create an environment that balances cognitive challenge with emotional support in order to foster greater learning enjoyment. Emotional support becomes particularly important when students face academic pressure or external evaluation. If students’ emotional needs are not addressed, their motivation may decline. Therefore, teachers should focus not only on students’ academic performance but also on providing encouragement and constructive feedback to help students remain confident and engaged in their learning. While the form of emotional support may vary across disciplines, it remains a critical factor in enhancing students’ learning enjoyment and supporting their academic development.

## 6 Conclusion

This study investigates how learning engagement, curiosity, and classroom experience collectively influence students’ learning enjoyment, offering a detailed examination of the complex interactions among these factors. The analysis reveals that learning engagement has the most substantial effect on learning enjoyment, with a regression coefficient of 0.68. This suggests that students’ active participation plays a central role in shaping their learning experience. However, the relationship between engagement and enjoyment is not straightforward. Learning enjoyment does not solely rely on students’ participation; rather, it is influenced by the classroom environment and the support provided by teachers. For engagement to result in heightened enjoyment, it must be paired with a challenging yet supportive classroom atmosphere. Thus, educators should prioritize fostering classroom interactions and ensuring emotional engagement to maximize the benefits of student participation. Curiosity also contributes significantly to learning enjoyment, with a regression coefficient of 0.63. While its influence is somewhat less pronounced than that of engagement, curiosity still plays a vital role in stimulating students’ learning interest. This suggests that students’ intrinsic desire to explore new knowledge is a key factor in their learning enjoyment, yet its effect is contingent on the complexity and stimulating nature of the tasks they encounter. When tasks lack challenge or fail to engage students, curiosity may not be fully activated, limiting the potential for increased enjoyment. Educators should focus on designing stimulating and thought-provoking tasks that not only captivate students’ curiosity but also enhance their enjoyment of learning. Classroom experience stands out as the most influential factor, with a regression coefficient of 0.70, underscoring the pivotal role that classroom environment and teacher-student interactions play in promoting learning enjoyment. A positive classroom experience can significantly boost students’ emotional engagement, directly enhancing their interest and enjoyment in learning. Classroom experience encompasses not only students’ perceptions of the physical learning space but also teacher-student relationships, classroom atmosphere, and emotional support. These elements collectively shape how students emotionally respond to learning, highlighting the importance of emotional support in the classroom. The study also explores the moderating effects of academic discipline and gender on learning engagement and enjoyment. The relationship between learning engagement and enjoyment is more pronounced among students in science disciplines, where the structured nature of tasks and challenges likely foster greater engagement. In contrast, gender has a minimal effect on engagement, suggesting that the impact of gender on students’ learning behaviors has diminished in modern educational contexts. This implies that educational strategies should focus more on addressing the individual needs of students rather than relying on generalized assumptions based on gender. In summary, this study offers valuable insights into how the interactive dynamics of learning engagement, curiosity, and classroom experience can enhance learning enjoyment. Educational practices should consider the specific needs of different academic disciplines, as well as the emotional and cognitive needs of students, to create a balanced and supportive learning environment. By aligning teaching strategies with these insights, educators can foster greater student motivation, engagement, and overall academic success.

## 7 Limitations and future directions

This study demonstrates the significant impact of learning engagement, curiosity, and classroom experience on students’ learning enjoyment while also identifying several limitations that provide valuable directions for future research. First, while the study employs a cross-sectional design that establishes correlations between variables, it does not allow for the determination of causal relationships. The correlation between learning engagement and learning enjoyment suggests that active participation in learning has a strong positive influence on students’ enjoyment; however, the long-term effects of this relationship remain unclear. Future studies should therefore adopt longitudinal or experimental research designs to explore how learning engagement evolves over time and how it subsequently influences learning enjoyment, thereby providing deeper insights into the causal mechanisms underlying these relationships. Another limitation of this research lies in its reliance on a purely quantitative research approach. Although questionnaire-based quantitative analysis is effective in identifying statistical relationships among variables, it may not fully capture the complexity of students’ learning experiences and psychological processes in real educational contexts. Future studies could therefore incorporate qualitative research methods, such as interviews, classroom observations, or mixed-method designs, to gain richer insights into students’ perceptions, emotions, and behavioral dynamics during the learning process. Such qualitative evidence would complement the quantitative findings and contribute to a more comprehensive understanding of how learning engagement, curiosity, and classroom experiences influence learning enjoyment. In addition, the sample used in this study is primarily drawn from specific regions and academic disciplines, which may limit the generalizability of the findings. Although the moderating effect of academic discipline on learning engagement is significant, students from different regions and educational systems may display different patterns of learning motivation and classroom experiences. Expanding the sample to include a more diverse range of cultural and disciplinary backgrounds would therefore enhance the external validity and generalizability of the results. Furthermore, in addition to the variables examined in this study, psychosocial factors such as self-efficacy, emotional regulation, and social support may also play important roles in shaping learning enjoyment. Future research could integrate these variables into the analytical model to better understand how they interact with learning engagement, curiosity, and classroom experience. Finally, although classroom experience was identified as a significant predictor of learning enjoyment, the present study does not directly examine how specific teaching strategies or instructional designs influence students’ enjoyment of learning. Future research could therefore design intervention-based studies to investigate how different teaching methods, classroom structures, and emotional support mechanisms affect students’ engagement and enjoyment, thereby providing more practical guidance for educators seeking to enhance learning experiences in higher education.

## Data Availability

The raw data supporting the conclusions of this article will be made available by the authors, without undue reservation.
