# Correction: Why do students lose the joy of learning? Evidence from engagement, curiosity, and classroom experience

**DOI:** 10.3389/fpsyg.2026.1860975

**Published:** 2026-05-01

**Authors:** Linqiang Wang, Xiaohuan Chen, Lu Sun, Chunming Chen

**Affiliations:** 1 School of Tourism, Shandong Women's University, Jinan, Shandong, China; 2 College of International Studies, Southwest University, Chongqing, China; 3 Accounting School, Nanfang College, Guangzhou, China; 4 School of Food and Health, Guangzhou City Polytechnic, Guangzhou, Guangdong, China

**Keywords:** classroom experience, curiosity, learning engagement, learning enjoyment, Self-Determination Theory, social cognitive theory, structural equation modeling, Task Value Theory

Author Chunming Chen was erroneously assigned as corresponding author. The correct corresponding author is Lu Sun.

The original version of this article has been updated.

